# Proteomic Profiling of EUS-FNA Samples Differentiates Pancreatic Adenocarcinoma from Mass-Forming Chronic Pancreatitis

**DOI:** 10.3390/biomedicines13092199

**Published:** 2025-09-08

**Authors:** Casandra Teodorescu, Ioana-Ecaterina Pralea, Maria-Andreea Soporan, Rares Ilie Orzan, Maria Iacobescu, Andrada Seicean, Cristina-Adela Iuga

**Affiliations:** 1 Department of Gastroenterology, “Prof. Dr. Octavian Fodor” Regional Institute of Gastroenterology and Hepatology, 400394 Cluj-Napoca, Romania; casandra.teodorescu@yahoo.ro (C.T.);; 2 Department of Internal Medicine, Faculty of Medicine, “Iuliu Hațieganu” University of Medicine and Pharmacy, Victor Babeș Str., No. 8, 400012 Cluj-Napoca, Romania; 3 Personalized Medicine and Rare Diseases Department, MEDFUTURE—Institute for Biomedical Research “Iuliu Hațieganu” University of Medicine and Pharmacy, Louis Pasteur Street 6, 400349 Cluj-Napoca, Romania; 4 Department of Pharmaceutical Analysis, Faculty of Pharmacy, “Iuliu Hațieganu” University of Medicine and Pharmacy, Louis Pasteur Street 6, 400349 Cluj-Napoca, Romania

**Keywords:** chronic pancreatitis, pancreatic ductal adenocarcinoma, mass-forming pancreatitis, EUS-FNA, label-free proteomics, mass spectrometry

## Abstract

**Background/Objectives:** Mass-forming chronic pancreatitis (MFP) and pancreatic ductal adenocarcinoma (PDAC) can present with overlapping radiological, clinical, and serological features in patients with underlying chronic pancreatitis (CP), making differential diagnosis particularly challenging. Current diagnostic tools, including CA19-9 and endoscopic ultrasound (EUS) imaging, often lack the specificity needed to reliably distinguish between these conditions. The objective of this study was to investigate whether the proteomic profiling of endoscopic ultrasound-guided fine-needle aspiration (EUS-FNA) samples could provide molecular-level discrimination between MFP and PDAC in patients with CP. **Methods**: Thirty CP patients with solid pancreatic lesions were prospectively enrolled: 15 with histologically confirmed PDAC and 15 with MFP. Traditional diagnostic parameters, including CA19-9 levels and EUS characteristics, were recorded but found insufficient for differentiation. EUS-FNA samples were analyzed using label-free mass spectrometry. A total of 928 proteins were identified in PDAC samples and 555 in MFP samples. Differential abundance analysis and pathway enrichment were performed. **Results**: Overall, 88 proteins showed significant differential abundance between PDAC and MFP samples, of which 26 met stringent statistical thresholds. Among these, Carboxylesterase 2 (CES2), Carcinoembryonic Antigen-Related Cell Adhesion Molecule 1 (CEACAM1), Lumican (LUM), Transmembrane Protein 205 (TMEM205), and NAD(P)H Quinone Dehydrogenase 1 (NQO1) emerged as key discriminatory proteins. Pathway enrichment analysis revealed distinct biological processes between the groups, including mitochondrial fatty acid β-oxidation, Rho GTPase signaling, and platelet degranulation. **Conclusions**: Proteomic signatures derived from EUS-FNA samples offer a promising molecular approach to distinguish inflammatory pseudotumoral lesions from malignant pancreatic tumors in CP patients. This minimally invasive strategy could enhance diagnostic accuracy where current methods fall short. Further validation in larger, multicenter cohorts is warranted to confirm these findings and evaluate their clinical applicability.

## 1. Introduction

The differential diagnosis of solid pancreatic masses includes a broad spectrum of malignant and benign entities. Malignancies such as pancreatic ductal adenocarcinoma (PDAC), neuroendocrine tumors, and metastases must be distinguished from benign conditions like autoimmune pancreatitis and mass-forming chronic pancreatitis (MFP) [1]. Approximately 10% of patients with chronic pancreatitis (CP) develop inflammatory pseudotumoral lesions, particularly in the pancreatic head, which can mimic the imaging and clinical presentation of PDAC [2]. The situation is further complicated by the fact that CP is an established risk factor for PDAC, with a reported 16-fold increased risk in the first two years following diagnosis and a 7.9-fold risk within five years [3].

Accurate distinction between benign inflammatory masses and malignant tumors is critical to guide appropriate management and avoid both unnecessary surgical resections and missed cancer diagnoses. Endoscopic ultrasound (EUS) is the primary imaging modality for pancreatic lesion evaluation due to its superior resolution. However, a meta-analysis has shown that EUS-guided fine-needle aspiration (EUS-FNA) exhibits markedly reduced sensitivity in patients with CP compared to those without (65.3% vs. 91.5%) [2]. This decreased performance reflects the technical and pathological challenges associated with sampling fibrotic and inflamed pancreatic tissue. To improve tissue acquisition, EUS-guided fine-needle biopsy (EUS-FNB) has gained favor over EUS-FNA for evaluating pancreatic masses [1]. Even so, its diagnostic sensitivity ranges from 85% to 88% in differentiating MFP from PDAC [4], and false negatives remain a concern, especially in patients with severe fibrosis or poorly defined lesions [5]. Furthermore, lesion size and typical CP features—such as lobularity without honeycombing—may reduce diagnostic accuracy in small masses [6]. Advancements in molecular diagnostics have improved sensitivity in some clinical scenarios. For instance, the sequencing of EUS-obtained samples has shown a success rate of approximately 84%, with KRAS mutation detection in 86% of malignant lesions [7]. However, this still leaves a substantial proportion of indeterminate cases, especially in the context of MFP and CP, where inflammatory changes may obscure or mimic malignancy.

Proteomics presents a powerful alternative by enabling large-scale investigation of protein expression, structure, and function. Mass spectrometry-based profiling allows proteomic analyses to identify disease-specific protein signatures, potentially surpassing the limitations of cytology and single-gene mutation testing—even in challenging malignancies such as PDAC [8]. While carbohydrate antigen 19-9 (CA19-9) remains the only validated biomarker for PDAC, its poor sensitivity and specificity—especially in early disease or in the presence of biliary obstruction—limit its diagnostic utility [9]. An elevated level of CA19-9 was reported in diabetes or cholestasis as benign condition as well as other malignancies such as gastrointestinal cancers [10]. Blood-based proteomic panels combining CA19-9 with apolipoproteins have demonstrated improved discrimination between PDAC and benign conditions [11] but remain investigational.

While proteomic approaches have advanced the differentiation of CP and PDAC, no proteomic biomarker has yet been validated for distinguishing MFP from PDAC. Previous studies using mass spectrometry-based platforms have focused on differentiating CP from PDAC using various biological matrices including pancreatic tissue and pancreatic juice and blood derived samples [12,13,14]—to identify differentially abundant proteins and potential biomarkers for diagnosis or patient stratification [15]. For example, Crnogorac–Jurcevic et al. [16] and Pan et al. [17] reported several proteins with discriminatory power using pancreatic tissue specimens from patients with chronic pancreatitis and PDAC. These included UHRF1, ATP7A, and AOX1 associated with epigenetic regulation, metabolic adaptation, immune evasion, and altered trafficking while collagen alpha-1(XIV) chain (COL14A1), lumican (LUM), and versican (VCAN) involved in matrix organization, remodeling, and cell–matrix interactions. Shen et al. [18] identified tumor-associated proteins—including ANXA4, PPIA, CTSD, LGALS1, and others—linked to inflammation, metabolic adaptation, and cellular stress.

Most proteomic studies have been performed on surgically resected pancreatic tissue [19,20,21,22,23], which restricts their clinical applicability, particularly in patients with unresectable disease or ambiguous lesions. Several proteins—such as DPC4, IMP3, Maspin, Mesothelin, p53, S100A4, S100P, and VHL—have shown potential as differential markers [22,23,24,25] in the context of identifying and distinguishing PDAC from other pancreatic lesions or benign tumors, but these findings have not been adequately validated yet in less invasive biopsy samples like EUS-FNA, compared to resected specimens obtained after surgical removal. Notably, Souche et al. [26] employed mass spectrometry-based proteomic profiling on EUS-FNA samples from patients with suspected pancreatic lesions, including PDAC and benign conditions such as chronic pancreatitis. They proposed a 19-protein panel—comprising markers like AGR2, PIGR, CYP2S1, MYH14, cytokeratins (KRT7, KRT18, KRT19), annexins (ANXA2–4), and TIMP1—to distinguish PDAC from non-malignant lesions. Collectively, these proteins are functionally linked to ER stress adaptation, cytoskeletal remodeling, metabolic reprogramming, and matrix interaction.

To our knowledge, no prior research has systematically profiled the proteome of EUS-FNA-derived samples to distinguish PDAC from MFP in the context of CP. Given the widespread clinical use of EUS-FNA and the diagnostic uncertainty associated with pancreatic masses in CP, there is a clear need for adjunct molecular tools that can increase diagnostic precision. Proteomic profiling of EUS-FNA specimens may fulfill this role by identifying specific protein markers capable of differentiating inflammatory from malignant lesions at a molecular level.

This study presents an exploratory analysis to identify molecular pathways and potential biomarkers distinguishing MFP from PDAC using MS-based proteome profiling of EUS-FNA samples.

## 2. Materials and Methods

### 2.1. Patient Cohort and Clinical Characterization

This prospective cohort study was conducted at the Regional Institute of Gastroenterology and Hepatology, Cluj-Napoca, Romania—a tertiary referral center—between January 2023 and April 2024. Written informed consent was obtained from all participants in accordance with the Declaration of Helsinki. The study protocol was approved by the Institutional Ethics Committee (Approval no. 1237, dated 27 January 2021). Eligible participants included patients with a confirmed diagnosis of CP and a solid pancreatic lesion identified via endoscopic ultrasound (EUS). Patients were excluded if they had coagulation abnormalities (international normalized ratio [INR] > 1.5 or platelet count < 50,000/μL), biliary metallic stents, duodenal obstruction, or tumors with a cystic component exceeding 20%.

For each patient, clinical and imaging data were collected, including medical history, EUS features (lesion location, vascularity, and size), contrast-enhanced EUS (CH-EUS) characteristics, and histopathological diagnosis. Descriptive statistics were used to summarize the cohort. Data were expressed as mean ± standard deviation (SD) for normally distributed continuous variables or as median with interquartile range (IQR) for non-normal distributions while frequencies and proportions were used to summarize categorical variables. Comparisons between the MFP and PDAC groups were performed using the two-sample t-test for continuous variables (e.g., age, lesion size), under the assumption of equal variances. Statistical significance was set at *p* < 0.05, with Bonferroni correction applied for multiple comparisons. For categorical variables such as sex, laboratory findings (e.g., elevated CA19-9, cholestasis, biliary retention, hepatocytolysis), and CP features, Yates’ corrected chi-squared test was used. All statistical analyses were conducted using IBM SPSS Statistics for Windows, version 25.0 (IBM Corp., Armonk, NY, USA).

### 2.2. Endoscopic Ultrasound-Guided Fine-Needle Aspiration (EUS-FNA) Technique and Sample Collection

All EUS-FNA procedures were performed using a linear-array echoendoscope (GF-UCT 180 AL5; Olympus, Tokyo, Japan) in conjunction with a Hitachi Arietta 850 ultrasound processor. Standard 22-gauge needles (EchoTip, Boston Scientific, Marlborough, MA, USA) were used for tissue acquisition. Procedures were carried out by a single experienced endoscopist (A.S.) under intravenous sedation with either midazolam or propofol.

A transduodenal approach was employed for lesions located in the pancreatic head or uncinate process, while transgastric access was used for lesions in the pancreatic body or tail. Prior to biopsy, the endosonographic features of CP were evaluated according to the Rosemont criteria [27]. When signs of CP were present, further assessment included lesion size and location, diameter of the main pancreatic duct and evidence of local (e.g., vascular involvement) or extended invasion (e.g., regional lymph nodes, left hepatic lobe metastases, ascites). Once the needle was advanced into the lesion, the stylet was removed, and a combination of fanning and suction technique was used for sampling. Three needle passes were performed for histopathological diagnosis, and an additional pass was made specifically for proteomic analysis. Samples for proteomics were placed in 2 mL of phosphate-buffered saline and stored at −80 °C until further processing. All patients were monitored for at least 8 h post-procedure to detect any complications.

Final diagnoses were based on histopathological evaluation of EUS-FNA specimens. Patients lacking definitive histological confirmation were excluded from the study. Those with negative cytology for malignancy were followed clinically, with serial assessments including CA19-9, abdominal ultrasound at 3, 6, 9, and 12 months, and computed tomography (CT) scans at 3 and 6 months after study inclusion.

### 2.3. Label Free Mass Spectrometry Measurement and Data Analysis

#### 2.3.1. Sample Preparation for Proteome Profiling Through Mass Spectrometry

Fine needle aspiration samples were thawed and centrifuged at 15,000× *g* for 20 min at 4 °C. The resulting cell pellet was washed once with PBS and solubilized in 8 M urea/2 M thiourea (UT) buffer. The complete sample preparation protocol has been thoroughly described elsewhere [28]. Briefly, protein extraction in UT buffer was enhanced by sonication. After a second centrifugation step (15,000× *g*, 20 min, 4 °C), total protein content in the supernatant was determined using the micro-Bradford assay (Bio-Rad Laboratories, Munich, Germany). A sample aliquot corresponding to 2 µg of total protein was subjected to reduction and alkylation using dithiothreitol (5 mM final concentration, 1 h at 60 °C) and iodoacetamide (40 mM final concentration, 15 min at 37 °C), respectively. Proteolytic digestion was performed overnight at 37 °C using trypsin (Sigma-Aldrich, St. Louis, MO, USA) at a 1:50 enzyme-to-substrate ratio. Digestion was terminated by acidifying the samples, followed by peptide purification using ZipTip μC18 pipette tips (Millipore-Sigma, Burlington, MA, USA). The purified peptides were dried in a vacuum concentrator (Thermo Scientific, Waltham, MA, USA) and reconstituted in 20 µL of 0.1% formic acid in a 2:98 (*v*/*v*) acetonitrile/water solution prior to injection.

#### 2.3.2. Mass Spectrometry Data Acquisition and Raw Data Processing

Data acquisition and primary processing were performed as previously described [28,29]. Briefly, for each run, 3 µL of sample (corresponding to 300 ng of protein on-column) was injected onto a reversed-phase Acquity UPLC M-Class Symmetry C18 trap column (180 µm × 20 mm, 5 µm particle size; Waters Corp, Milford, MA, USA) at a flow rate of 5 µL/min in 99% solvent A (0.1% formic acid in water). After a 2-min trapping and wash phase, peptide separation was carried out on a nanoAcquity UPLC M-Class T3 column (75 µm × 150 mm, 1.8 µm particle size; Waters, Wexford, Ireland) using a 45-min multistep concave gradient from 5% to 85% solvent B (0.1% formic acid in acetonitrile) at 0.3 µL/min. The column temperature was maintained at 55 °C. Peptides were ionized in positive mode using a nanoESI source and analyzed with a SYNAPT G2-Si HDMS instrument (Waters Corporation, Wilmslow, UK) operated in high-definition ion mobility (HDMSe) mode as described here [28]. Spectra were acquired across an *m*/*z* range of 50–2000 with a scan time of 0.5 s in resolution mode. Glu-1-fibrinopeptide B (Glu-Fib, 100 fmol/µL) served as the LockMass reference.

Raw data were processed using Progenesis QIP v4.2 (Nonlinear Dynamics, Waters Corporation) as detailed in [28]. Protein identification was performed using a target-decoy UniProtKB/Swiss-Prot human database (20,361 entries, downloaded January 2022) with the following parameters: enzyme specificity for trypsin; maximum one missed cleavage; carbamidomethylation as a fixed modification while methionine oxidation as a variable modification. The FDR threshold was set at <1%. Ion match requirements implemented were as follows: (i) minimum 1 fragment ion matches per peptide ion; (ii) 3 fragment ions matched per protein identification; and (iii) at least 1 peptide match per protein identification. Peptides with a sequence length of less than six amino acids and a mass error of more than 20 ppm were removed. Protein quantification was based on non-conflicting peptide intensities, resulting in 1039 total proteins. The final protein list was exported for down-stream analysis. The overall FDR at the dataset level was calculated to be 3.56%.

#### 2.3.3. Data Analysis

MetaboAnalyst 6.0 (accessed on 17 May 2025) was used for post-processing. Data normalization missing value imputation parameters and differential expression analysis followed those previously described in [28]. Proteins were considered differentially expressed if they met both a *p*-value threshold ≤ 0.05 and an absolute fold change (|FC|) ≥ 1.2. Graphical visualizations, including volcano plots, were generated within the same platform.

Functional analysis was conducted using Panther database v19 (https://pantherdb.org/, accessed on 30 July 2025) using the following gene ontologies: Molecular function (MF) and Biological process (BP) GO ontologies and Panther Protein Class (PC) annotation database.

ClueGO (v2.5.10) and CluePedia tools (v1.5.10) [30] within Cytoscape (v3.10.3) [31] were used for conducting pathway enrichment analysis, referencing the Reactome database (version 25 May 2022, 2535 terms, 10,882 unique genes). Pathways were defined as significantly enriched if they included at least three mapped proteins and had a corrected *p*-value (Benjamini–Hochberg) ≤ 0.005 as described here [28]. Supplementary Data (e.g., average FC, highest condition, number of genes/term) was connected onto the networks and visualized using Omics Visualizer (v1.3.1). Tissue specificity and distribution of the selected proteins along with other relevant data were extracted from Human Protein Atlas database (version 24; available online at proteinatlas.org [32]).

Protein–protein interaction (PPI) networks and enrichment analyses were per-formed using the STRING database v12 (https://string-db.org, accessed on 25 June 2025) as described previously [33]. For improved clarity, gene names were used throughout the manuscript instead of protein accession IDs.

## 3. Results

### 3.1. Patient Characteristics

Out of a total of 30 patients who met the inclusion criteria, half were ultimately diagnosed with histopathologically PDAC, while the other half were diagnosed with MFP. The patients’ demographics, blood tests, as well as the EUS features are listed in Table 1.

### 3.2. Proteomics Profiles Analysis

A total of 1001 proteins were identified based on quantitative data derived from 12,855 peptide ions using Progenesis QIP. Of the initial 30 samples, 28 were retained for post-processing analysis; two samples (one from each group) were excluded due to insufficient alignment scores in Progenesis QIP. Within the final dataset, 928 proteins were identified in PDAC samples, 555 proteins in MFP samples, with 453 proteins shared between the two groups. Further data processing was conducted using MetaboAnalyst 6.0, as detailed in the Materials and Methods section.

#### 3.2.1. Differentially Abundant Proteins Distinguishing the Pancreatic Lesions

Differential abundance analysis identified 90 proteins with statistically significant differences in abundance between the MFP and PDAC groups (*p* ≤ 0.05). Of these, 88 proteins also met a fold change threshold of |FC| ≥ 1.2 (Table S1). When applying a more stringent criterion (*p* ≤ 0.01 and |FC| ≥ 1.2), 26 proteins remained significantly differentially abundant (Table S1, Figure S1). Based on the Human Protein Atlas (HPA), 37 of these DAPs demonstrate pancreatic tissue specificity. Notably, 51 of the identified proteins exhibit high or medium expression in exocrine pancreatic cells, and 33 are found in endocrine cell compartments (Table S2).

**Figure 1:**
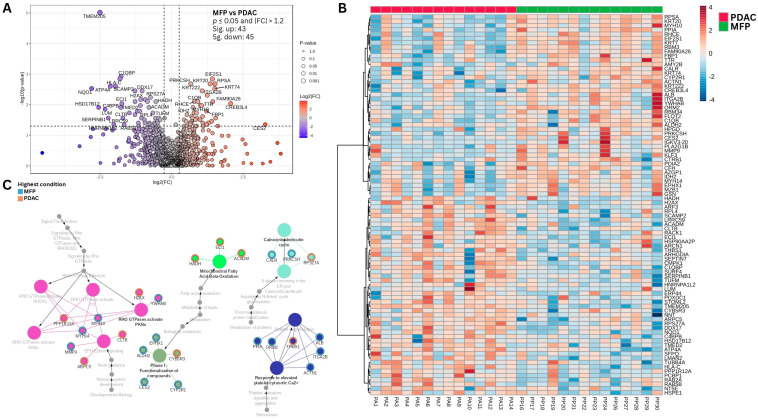
Analysis and visualization of differentially abundant proteins and pathway enrichment in MFP and PDAC. (**A**) Volcano plot illustrates differentially abundant proteins (t-test, independent unequal variance, *p* ≤ 0.05 and |FC| > 1.2) comparing the MFP group to the PDAC group. Proteins with significantly higher abundance in MFP are highlighted in red, those with higher abundance in PDAC are shown in blue, and proteins without significant differential abundance are depicted in gray. Gene names are labeled for all significant proteins; (**B**) Heatmap of differentially abundant proteins (unpaired *t*-test, unequal variance, *p* ≤ 0.05), clustered using Euclidean distance metrics; (**C**) Overrepresentation analysis (ORA) results generated using Cytoscape ClueGO and Cluepedia with the Reactome pathway database; Each node in the network represents a Reactome pathway, with node color indicating functional groupings based on shared protein constituents. Gray nodes correspond to higher-order hierarchical pathways that organize related pathways according to the Reactome ontology. Individual differentially abundant proteins are also represented as nodes, labeled by their gene names. A colored halo surrounding each protein node denotes the group in which the protein is most abundant: orange for PDAC and blue for MFP.

Analysis and visualization of differentially abundant proteins are presented in Figure 1A,B. Over-representation analysis using ClueGO and CluePedia revealed several significantly enriched Reactome pathways among the differentially abundant proteins (Figure 1C, Table S3). Notably, multiple pathways related to Signaling by Rho GTPases (R-HSA:194315) were enriched, including RHO GTPases-activate ROCKs, activate PAKs, and activate PKNs. Proteins contributing to this enrichment included Myosin-10 (MYH10), Protein phosphatase 1 regulatory subunit 12A (PPP1R12A), Actin-related protein 2/3 complex subunit 5 (ARPC5), Histone H2AX (H2AX), and Myosin-14 (MYH14). Additional enriched pathways included EPH–Ephrin signaling (R-HSA:2682334), supported by the presence of Ephrin type-A receptor 1 (EPHA1), and Mitochondrial Fatty Acid β-Oxidation (R-HSA:77289), involving proteins such as Medium-chain specific acyl-CoA dehydrogenase (ACADM), Hydroxyacyl-Coenzyme A dehydrogenase (HADH), and Enoyl-CoA hydratase, mitochondrial (ECH1). Pathways related to Platelet Degranulation (R-HSA:114608) were also significantly enriched, including proteins such as Thrombospondin-1 (THBS1), Albumin (ALB), Integrin alpha-IIb (ITGA2B), and Actin, alpha cardiac muscle 1 (ACTN1). Similarly, the Calnexin/Calreticulin Cycle (R-HSA:901042) was enriched in this dataset through proteins including Calreticulin (CALR), Protein kinase C substrate 80K-H (PRKCSH), and 40S ribosomal protein S27a (RPS27A).

#### 3.2.2. Functional Classification and Enrichment Analysis of the Differentially Abundant Proteins

According to the functional classification based on GO MF, most DAPs have binding (GO:0005488, 43.0%) and catalytic activity (GO:0003824, 30.4%). Specifically, these proteins are primarily involved in binding to proteins (GO:0005515), protein complexes (GO:0044877), and small molecules (GO:0036094), or exhibit catalytic functions such as hydrolase (GO:0016787) and oxidoreductase activity (GO:0016491). Notably, functional classification of DAPs using the PANTHER GO BP ontology revealed their significant involvement in cellular processes (GO:0009987, 36.2%) and metabolic processes (GO:0008152, 20.3%). Within the cellular processes category, cellular metabolic processes (GO:0044237) and cellular component organization (GO:0071840) were the most represented subcategories. In addition, DAPs were also associated with other biological processes such as cellular localization (GO:0051641), cellular response to stimuli (GO:0051716), actin filament-based processes (GO:0030029) or vesicle-mediated transport (GO:0016192). PANTHER PC functional classification revealed DAPs predominantly involved in metabolite interconversion (PC00262, 22.2%), underscoring their roles as oxidoreductases and hydrolases. Additionally, several DAPs were classified as cytoskeletal proteins (PC00085, 11.1%) or were associated with RNA metabolism (PC00031, 6.9%) and defense mechanisms (PC00090, 6.9%) (Figure S1C).

**Figure 2:**
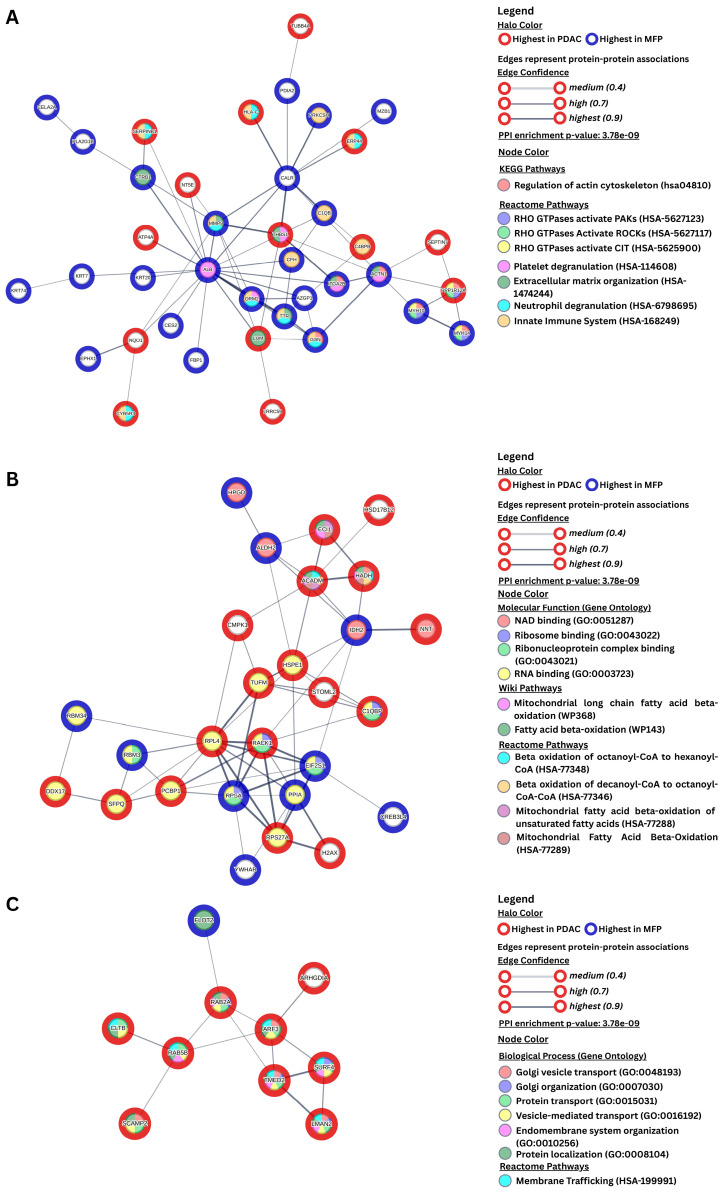
Protein–protein interaction (PPI) networks of differentially abundant proteins between MFP and PDAC, generated using STRING and annotated with functional enrichment data. (**A**) Network enriched for pathways related to cytoskeleton regulation and immune system function; (**B**) network enriched for mitochondrial metabolic pathways and RNA/protein binding functions; (**C**) network associated with Golgi organization and vesicle-mediated transport. Node halo color indicates condition-specific abundance (red = highest in PDAC; blue = highest in MFP). Node fill color denotes pathway or functional annotation. Edge thickness represents confidence of protein–protein association.

Protein–protein interaction (PPI) and functional enrichment analyses were performed using the STRING database (https://string-db.org/, accessed 25 June 2025). The analysis revealed that differentially abundant proteins (DAPs) exhibited a significantly higher degree of interaction than would be expected for a random set of proteins of similar size and connectivity (PPI enrichment *p*-value = 3.78 × 10^−9^) (Figure S2, Table S4). The resulting PPI network was partitioned into three clusters using the k-means clustering algorithm (Figure 2A–C). Functional enrichment analysis was conducted using STRING’s built-in enrichment tools, incorporating multiple annotation sources including KEGG, Reactome, WikiPathways, and Gene Ontology (FDR ≤ 0.05, minimum of three proteins per pathway). The enriched pathways were consistent with results obtained via ClueGO/CluePedia analysis and included signaling pathways such as those involving RHO GTPases, platelet degranulation (HSA-114608), neutrophil degranulation (HSA-6798695), and mitochondrial fatty acid β-oxidation (HSA-77289).

Cluster 1 (Figure 2A) was enriched for proteins associated with the regulation of the actin cytoskeleton (HSA-04810), signaling by RHO GTPases, extracellular matrix organization (HSA-1474244), and immune-related functions. The proteins in this cluster formed a highly connected subnetwork related to cytoskeletal regulation, immune defense, and extracellular matrix remodeling. Cluster 2 (Figure 2B) included proteins enriched in pathways related to mitochondrial fatty acid β-oxidation (HSA-77288, WP368) and RNA metabolism, including RNA and ribosome binding (GO:0003723, GO:0043021). Cluster 3 (Figure 2C) comprised proteins functionally enriched in vesicle-mediated transport and membrane trafficking (HSA-199991), including Golgi vesicle transport (GO:0048193), protein localization (GO:0008104), and related vesicular transport processes.

## 4. Discussion

Differentiating MFP from PDAC remains a major clinical challenge due to overlapping clinical, serological, and imaging features, often leading to misdiagnosis, delayed treatment, or unnecessary surgery [1,3,5]. While MFP is a benign inflammatory process often arising in chronic or autoimmune pancreatitis, PDAC is a malignant epithelial neoplasm with poor prognosis. In our cohort, neither EUS characteristics nor conventional biomarkers such as CA19-9 effectively distinguished between the MFP and PDAC, emphasizing the need for reliable and discriminative diagnostic tools.

To our knowledge, this is the first study to specifically isolate MFP as a clinical subtype of CP and to employ proteomic profiling of EUS-FNA-derived tissue samples with the aim of uncovering molecular signatures (Tables S1 and S2) that could support more accurate differentiation of the two conditions.

### 4.1. Cytoskeletal and Structural Protein Dynamics

A central finding of this study was the differential regulation of cytoskeletal and structural proteins between MFP and PDAC. Several proteins classically linked to pancreatic function (e.g., ACTN1, GSN, MYH10, ARHGDIA, and SEPTIN7) were differentially abundant (Tables S3 and S4). ACTN1 showed higher abundance in our MFP group, opposed with reports from Crnogorac–Jurcevic et al. [16] where it was enriched in PDAC versus both chronic pancreatitis and normal pancreas. Previous study showed that ACTN1 is central to PDAC micropinocytosis, extracellular matrix processing, and chemotherapeutic uptake [34] facilitating cancer survival. Its higher abundance in MFP in our study may reflect maintenance of epithelial architecture and active regenerative remodeling in MFP. Other actin-related proteins, such as GSN and MYH10, were also upregulated in MFP suggesting active cytoskeletal reorganization in response to inflammation or regeneration. ITGA2B also showed higher abundance in MFP, further indicating enhanced cell–matrix adhesion in the non-malignant setting. Several cytokeratins were also enriched in MFP including KRT7, KRT20, KRT74 and KRT222. While KRT7 can be upregulated in PDAC and is linked to metastatic potential and extracellular matrix remodeling in advanced disease, its increased abundance in MFP may instead reflect a role in maintaining epithelial integrity and resist dedifferentiation [35]. Notably, the increased abundance of KRT74, KRT20, KRT222, MYH14 and ITGA2B may reflect maintenance of epithelial architecture and active regenerative remodeling in MFP. Additionally, we found other abundant proteins in PDAC samples implicated in cytoskeletal dynamics, cell motility and tumor progression such as TUBB4A, SEPTIN7, ARHGDIA, THBS1, and PPP1R12A, similar to reports from the literature [36,37,38].

Taken together, the cytoskeletal signature of MFP appears to reflect epithelial preservation and remodeling, while PDAC exhibits a more invasive, contractile, pro-migratory cytoskeletal program consistent with tumor–stroma interaction and metastatic progression.

### 4.2. Transcriptional, RNA Processing, and Translational Signatures

Numerous differentially abundant proteins (DAPs) involved in transcriptional regulation, RNA processing, and translational control were identified between MFP and PDAC, highlighting key differences in biosynthetic and stress-adaptive responses. In MFP, several proteins associated with canonical protein synthesis were enriched, including RPSA and EIF2S. These may reflect an adaptive upregulation of protein biosynthesis in response to chronic tissue stress or regenerative signaling. RBM34, as a RNA-binding protein, and KLF3, as a transcription factor, were also found more abundant in MFP. They facilitate the proper regulation of gene expression and mRNA processing, which are essential for maintaining differentiated cell states and tissue integrity [39]. PPIA (cyclophilin A), a chaperone that facilitates protein folding and regulates transcription, was also found more abundant in MFP within our dataset. While typically linked to poor PDAC prognosis [40], its expression in MFP may instead signify a protective response to chronic stress or inflammation.

Several proteins, RPL4, RPS27A, PCBP1, HNRNPA1L2, and PDXDC1, involved in RNA metabolism and translation were significantly more abundant in PDAC, reflecting an upregulated translational state. The higher abundance of these proteins in PDAC has been previously reported [41,42,43,44] and associated with enhanced protein synthesis, dysregulated RNA metabolism, and metabolic reprogramming. Interestingly, DEAD-box helicase 17 (DDX17) was highly abundant in PDAC and was studied in the context of cancer initiation and progression. Moreover, it has been included previously in proteomic panels designed to differentiate PDAC patients from non-cancerous controls [45]. Furthermore, PDAC samples also showed higher abundance of H2AX, a DNA damage response protein. Previous reports have highlighted the phosphorylated form, γ-H2AX, in PDAC associating it with aggressive tumor biology and poor prognosis [46].

### 4.3. Metabolic Reprogramming and Redox Regulation

Differential expression analysis of metabolic proteins between MFP and PDAC underscores divergent energy demands and biosynthetic programs in these conditions. In MFP, signal transduction proteins such as YWHAB and FLOT2, along with CYP2R1 (involved in vitamin D metabolism), showed higher abundance. Transthyretin (TTR) was also elevated in MFP, perhaps indicating a less systemically catabolic state in a non- malignant disease. Interestingly, while serum TTR was typically reported to be elevated in PDAC compared to normal control [47], lower levels in PDAC have been linked to advanced disease and poor prognosis in resected PDAC [48]. Among proteins associated with metabolism and detoxification (CES2, ALDH2, HPGD, IDH2), CES2 was found to be more abundant in MFP compared to PDAC in our dataset. While often elevated in PDAC relative to normal control and associated with poor prognosis [49], its expression varies with tumor differentiation and microenvironmental factors [50]. These findings highlight the complex, context-dependent role of CES2 in pancreatic disease biology and therapeutic response.

PDAC samples demonstrated increased abundance of mitochondrial and redox-regulating proteins (HSPE1, ACADM, TUFM, STOML2, NNT, C1QBP) as well as factors involved in transcription, splicing, and protein biosynthesis (SFPQ, RPS27A, CMPK1). These findings suggest a coordinated upregulation of transcription, translation, and energy production, aligning with the well-established metabolic rewiring observed in cancer cells [36,51].

Among proteins elevated in PDAC, NQO1 and CYB5R3 stood out. NAD(P)H: quinone oxidoreductase 1 (NQO1), an antioxidant enzyme critical for maintaining redox homeostasis [52], is well-documented in PDAC. Previous immunohistochemical and transcriptomic studies report high NQO1 levels in pancreatic tumor tissues and cell lines, with markedly lower abundance in normal or benign pancreatic tissue NQO1 as a therapeutic target [53,54,55]. Functional studies support this potential: inhibition of NQO1 with dicumarol increased intracellular superoxide levels and suppressed pancreatic cancer cell growth in vitro [56]. Similarly, CYB5R3 is primarily involved in redox regulation, lipid metabolism, and detoxification was also enriched in PDAC in our study. While its tumor-suppressive role has been described in other cancers [57], conclusive data on its presence and role in pancreatic diseases remain lacking, warranting further investigation. Finally, HSD17B12, a key enzyme in lipid biosynthesis and steroid metabolism, was highly abundant in PDAC in our study. It has been reported upregulated in PDAC stem-like cells, supporting enhanced lipid biosynthesis—a metabolic hallmark of PDAC [58].

### 4.4. Endoplasmic Reticulum (ER) Stress and Proteostasis

Several differentially abundant proteins (DAPs) related to ER function and stress response were more abundant in MFP, potentially reflecting preserved ER homeostasis and cellular resilience under chronic inflammatory conditions. Among these, PRKCSH, a glucosidase II subunit involved in glycoprotein folding, was one of the lowest abundant proteins in PDAC in both the present and previous studies [16]. Reduced PRKCSH expression has been associated with impaired protein folding, increased ER stress [59] and anti-tumor immunity [60]. Similarly, CREB3L4, a transcription factor involved in ER stress response, emerged as one of the top DAPs enriched in MFP. While the broader CREB3 family plays known roles in cancer biology and ER homeostasis—and CREB1 is implicated in pancreatic cancer progression and β-cell differentiation—CREB3L4 remains largely unexplored in pancreatic disease and is not currently recognized as a biomarker or functional regulator in either PDAC or chronic pancreatitis [61]. Additionally, PDIA2, a member of the protein disulfide isomerase (PDI) family was found to be abundant in MFP. Members of the disulfide isomerase (PDI) family ensure proper folding of digestive enzymes and other secretory proteins, thereby maintaining proteostasis and preventing the accumulation of misfolded proteins that can trigger ER stress and cell injury [62] and may, in the context on MFP, be a marker of maintenance of functional acinar cells and ER homeostasis. RBM3, a cold-shock protein involved in RNA stability and stress adaptation, was also found to be more abundant in MFP. Although typically linked to stress adaptation and poor prognosis in PDAC [63], in the context of MFP it may instead reflect a regenerative response to inflammation.

In contrast, ER function and stress response proteins STOML2 and NNT were more abundant in PDAC. These have been previously implicated in mitochondrial remodeling, immune modulation and altered vesicle trafficking [64]. Their coordinated upregulation in PDAC reflects a shift toward tumor-supportive metabolic reprogramming and signaling adaptations. ERP44, a protein essential for proper folding and transport of proteins within the endoplasmic reticulum (ER), has not been extensively studied in PDAC, yet we found it more abundant in PDAC. Nishimura et al. [65] reported upregulation of ER stress and unfolded protein response pathways (which includes ERP44) in aggressive tumor phenotypes, suggesting a potential role in PDAC pathophysiology. Finally, TMEM205 was among the most highly abundant proteins in PDAC within this study. Transmembrane protein 205 (TMEM205) is one of several transmembrane proteins linked to PDAC prognosis and is involved in membrane trafficking, signaling, and tumor microenvironment modulation [66,67] highlighting their potential as both diagnostic and therapeutic targets. TMEM205 has been previously implicated in cisplatin resistance [68].

### 4.5. Immune Modulation and Tumor Microenvironment Signaling

Immune modulation emerged as a discriminatory feature between PDAC and MFP proteomes, reflecting their distinct immunopathological landscapes. Several proteins implicated in immune suppression and tumor microenvironment modulation were significantly more abundant in PDAC than MFP. Particularly, NT5E (CD73)—a key ectoenzyme that generates extracellular adenosine- promotes an immunosuppressive tumor microenvironment by inhibiting antitumor immune responses and facilitating metabolic crosstalk between cancer cells and cancer-associated fibroblasts. High CD73 expression in PDAC was associated with increased polyamine metabolism, tumor cell proliferation, migration, and the enrichment of immunosuppressive stromal elements [69]. Similarly, C1QBP involved in the mitochondrial function and immune regulation with consistent literature regarding its involvement in supporting tumor cell survival, modulating immune cell dynamics, and contributing to immune escape mechanisms [70,71] presented higher abundance in PDAC within this study. RAB proteins, CLTB, and SCAMP2 are central to vesicular trafficking and exosome biogenesis [72]. Their upregulation in PDAC enhances the secretion of extracellular vesicles and exosomes that can mediate intercellular communication within the tumor microenvironment, promoting stromal activation and immune suppression [73,74]. Compared to MFP, PDAc groups showed significantly higher abundance of HSP90AA2 (Heat Shock Protein 90 Alpha Family Class A Member 2). HSP90AA2 has been consistently reported highly abundant in PDAC compared to non-malignant pancreatic tissue, its increased abundance being associated with tumor progression, invasion, and metastasis. The HSP90α protein, encoded by HSP90AA2, is secreted by both tumor cells and tumor-associated macrophages, promoting epithelial–mesenchymal transition (EMT) and enhancing the invasive phenotype of PDAC cells [75,76]. In this study we also found other proteins involved in immune modulation such as C4BPB, HLA-C and SERPINB1 to be more abundant in PDAC than in MFP. While the roles of C4BPB, HLA-C, and SERPINB1 in cancer immunobiology have been increasingly recognized, direct evidence of their function in PDAC remains limited. Higher abundance of C4b-binding protein, particularly the alpha chain (C4BPA), has been associated with enhanced antitumor immunity in the PDAC tumor microenvironment [77,78]. HLA-C, a key molecule in antigen presentation, may also contribute to immune evasion in PDAC. Its upregulation has been linked to the expression of immune checkpoint inhibitors (e.g., PD-L1, PD-L2) and immunosuppressive cytokines, fostering an immunosuppressive microenvironment [79,80]. SERPINA1, particularly, acts as an oncogene, promoting invasion, metastasis, and proliferation via the PI3K/Akt/NF-κB pathway. Moreover, SERPINA1 was also associated with immune cell infiltration and immune checkpoint gene expression highlighting its role in shaping the immunosuppressive microenvironment [81,82].

The MFP proteome was enriched for several immune-related and plasma proteins more indicative of inflammatory infiltration and chronic immune activation rather than tumor-specific remodeling including C1QB, CFH, MZB1, IGKV3-20, ORM2, ALB, and AZGP1. Many of these proteins have previously been proposed as biomarker candidates for chronic pancreatitis. For instance, AZGP1 and ALB were highlighted as potential diagnostic markers in chronic pancreatitis [83] while ORM2 has been implicated in inflammatory pancreatic conditions [84]. Components of the immunoglobulin system, such as IGKV3-20 and MZB1, are typically associated with immune responses and inflammatory activity [85], underscoring their relevance to non-neoplastic pancreatic pathology. These proteins, rather than reflecting cancer-specific processes, are more indicative of immune infiltration and local inflammation [86], a characteristic of MFP. CEACAM1 was elevated in MFP in our study. While previously linked to early pancreatic neoplasia (PanIN) [87], its role in invasive PDAC remains debated, with studies reporting both upregulation [88,89,90] and loss of expression during progression [87,91]. Given its known function in immune regulation, elevated CEACAM1 in MFP likely reflects chronic inflammation rather than malignancy. These findings underscore its context-dependent role across the spectrum of pancreatic disease [92,93,94], an aspect which warrants further investigation in a study design better suited to resolving this complexity.

### 4.6. Integrative Interpretation and Promising Markers to Be Further Explored in Context of MFP and PDAC

The PPI network analysis further contextualizes the trends highlighted throughout the discussion, revealing three major functional axes that are distinctly programmed in MFP and PDAC: (i) the tumor–stroma interface, (ii) metabolic and translational control, and (iii) vesicle trafficking. The first network cluster (Figure 2A) included DAPs associated with extracellular matrix (ECM) and cytoskeletal remodeling, immune and complement activation, ER stress, protein folding, and pancreatic secretory functions. Notably, most of these DAPs were elevated in MFP, while only a small subset showed enrichment in PDAC. Hub proteins such as ALB, CALR, MMP9, ACTN1, and GSN were higher abundant in MFP suggesting active-matrix turnover, immune responsiveness, and preservation of secretory functions in chronic inflammation. Cluster 2 (Figure 2B) centers on mitochondrial metabolism, RNA processing, and translation. A considerable number of DAPs in cluster 2 were more abundant in PDAC, which may reflect an increased reliance on mitochondrial activity and biosynthetic processes (RPL4, RACK1, ACADM. In contrast, proteins more abundant in MFP (EIF2S1, PPIA, IDH2) may indicate a stress-adaptive or regulatory response linked to inflammation or redox balance. Overall, this network cluster captures a differential regulation of energy metabolism and translational machinery between the inflammatory and neoplastic settings, with PDAC potentially favoring biosynthetic activity and MFP showing signatures of metabolic adaptation to the inflammatory environment. On the other hand, cluster 3 (Figure 2C) encompassed vesicle trafficking and intracellular transport, with PDAC showing widespread upregulation of proteins involved in ER-to-Golgi transport (ARF3, RAB2A, TMED2), endocytosis (RAB5B, CLTB), and cargo recognition (SURF4, LMAN2). This reflects enhanced vesicular dynamics in PDAC. In contrast, MFP has limited representation in this module, except FLOT2, potentially reflecting a distinct role in membrane-associated immune or signaling processes.

Several proteins that were identified as particularly interesting based on their biological function, differential abundance, or potential relevance to MFP vs. PDAC biology are summarized in Table 2.

### 4.7. Study Limitations and Future Directions

This study has several limitations that should be acknowledged. Most notably, the statistical power and generalizability of the findings is limited due to the relatively low number of samples per group. To address patient variability and improve robustness, future studies should incorporate larger, prospective, and multi-center cohorts.

While our proteomic approach identified several differentially abundant proteins between MFP and PDAC, these findings remain preliminary and exploratory, the current results are intended to generate hypotheses and identify candidate proteins for further validation. While the proteomic findings provide novel and biologically plausible targets, confirmation using complementary analytical techniques and in external patient samples will be essential to substantiate the diagnostic potential of these markers.

A further limitation lies in disease specificity. While several DAPs emerged as potential biomarkers distinguishing MFP from PDAC, many of these proteins are not disease specific and may also be dysregulated in other conditions. Future studies should include broader disease comparisons to refine the specificity and translational potential of these markers.

It is also important to consider the impact of sampling techniques since the choice of endoscopic sampling technique can influence both the quantity and quality of the biological material retrieved, which in turn may affect the diagnostic yield and the reliability of proteomic profiling. Recent studies have shown that fine-needle biopsy may offer superior diagnostic performance compared to traditional fine-needle aspiration, particularly in terms of tissue architecture preservation and cellularity [95,96]. These methodological differences are especially relevant when downstream analyses such as proteomics require higher sample integrity. In this study, all samples were collected via EUS-FNA, which has known limitations in certain diagnostic contexts. Future work should consider evaluating the impact of sampling strategies to optimize the detection and validation of biomarkers.

Despite these limitations, the study also has significant strengths: it includes patients with MFP- a clinically challenging and understudied condition. The comparison of the proteomics signatures of MFP to PDAC provides valuable insights into the metabolic differences between these conditions. Another key strength lies in the use of endoscopic ultrasound-guided fine-needle aspiration samples for proteomic analysis as already portrayed in the first part of the discussion section. To our knowledge, this is the first study to apply high-throughput proteomics to this specific biological matrix, in the understudied chronic pancreatitis subgroup of MFP, using the described MS platform. This approach enhances the translational relevance of the findings supporting their future applicability in minimally invasive diagnostics. Planned studies by our group will aim to validate these findings, evaluate disease specificity, and explore the biological roles of less-characterized proteins such as HSD17B12 and CREB3L4.

## 5. Conclusions

This is the first study to specifically isolate MFP as a clinical subtype of CP and to employ proteomic profiling of EUS-FNA-derived tissue samples to distinguish it from PDAC. Our findings demonstrate feasibility and diagnostic potential.

Distinct proteomic signatures were identified, with PDAC showing enrichment in proteins related to chemoresistance, metabolic reprogramming, and vesicle trafficking, while MFP retained markers of cytoskeletal integrity and acinar function. The PPI network analysis revealed three distinct functional modules differentiating PDAC and MFP, namely, the tumor–stroma interface was dominated by proteins elevated in MFP, the metabolic and translational control cluster had enhanced biosynthetic and mitochondrial activity in PDAC and, the vesicle trafficking module was also largely upregulated in PDAC. These findings suggest spatially distinct biological adaptations that may influence tumor progression and therapeutic responses.

Although promising, further validation in larger cohorts and integration with other omics data are necessary. The use of EUS-FNA, while feasible, has known limitations in tissue quality and quantity, and future studies should explore alternative or complementary sampling techniques. In addition, evaluating the specificity of identified biomarkers against other pancreatic conditions will be essential for clinical translation.

This study lays important groundwork toward developing minimally invasive, molecular-based tools for improving diagnostic accuracy and clinical management in pancreatic disease.

## Figures and Tables

**Table 1:** Patients’ characteristics and EUS features.

Characteristic	PDAC (*n* = 15)	MFP (*n* = 15)	*p*-Value
Demographics			
Age, mean (SD) {range}	64.8 (9.59) {46–80}	57.6 (14.5) {30–81}	0.11
Male, *n* (%)	9 (60)	14 (93)	0.08
Blood tests			
Elevated CA19-9, *n* (%)	10 (67)	7 (47)	0.46
Cholestasis, *n* (%)	9 (60)	8 (53)	0.71
Biliary retention, *n* (%)	8 (53)	8 (53)	0.46
Hepatocytolysis, *n* (%)	8 (53)	8 (53)	1.00
EUS features			
Location, *n* (%)			
Head	10 (66)	11 (73)	
Uncinate process	1 (7)	3 (20)	
Neck	2 (13)	-	
Body	1 (7)	-	
Body-tail	1 (7)	-	
Tail	-	1 (7)	
Size, median (SD) {range}, cm	2.6 (0.54) {1.8–3.5}	2.75 (1.03) {1.5–4.5}	0.99
Features of chronic pancreatitis Major criteria, *n* (%)			
Hyperechoic foci with shadowing, *n* (%)	1 (7)	5 (33)	0.67
Main pancreatic duct calculi, *n* (%)	0	1 (7)	-
Lobularity with honeycombing, *n* (%)	6 (40)	10 (67)	0.33
Minor criteria, *n* (%)			
Irregular pancreatic duct, *n* (%)	9 (60)	11 (73)	0.54
Dilatated side branches, *n* (%)	0	0	-
Hyperechoic duct walls, *n* (%)	0	0	-
Cysts, *n* (%)	1 (7)	0	-
Main pancreatic duct dilatation, *n* (%)	8 (53)	9 (60)	0.71
Strands, *n* (%)	0	0	-
Hyperechoic foci without shadowing, *n* (%)	14 (93)	11 (73)	0.73
Lobularity without honeycombing, *n* (%)	10 (67)	4 (27)	0.40
Consistent with CP diagnosis, *n* (%)	1 (7)	5 (33)	0.17
Suggestive of CP, *n* (%)	14 (93)	10 (67)	0.17

Abbreviations: PDAC = pancreatic ductal adenocarcinoma, MFP = mass-forming chronic pancreatitis, SD = standard deviation, CA19-9 = carbohydrate antigen 19-9, EUS = endoscopic ultrasound, w/o = without.

**Table 2:** Key high-abundant proteins differentially expressed in MFP vs. PDAC.

GeneID (Protein)	Biological Function	Notes
MFP		
**CES2** Carboxylesterase 2	Drug-metabolizing enzyme involved in the hydrolysis of esters and amides	Top FC in MFP. May indicate active xenobiotic or detoxification processes in MFP. Not well studied in pancreatic tissue.
**CREB3L4** Cyclic AMP-responsive element-binding protein 3-like protein 4	ER-resident transcription factor involved in unfolded protein response	Top FC in MFP. Poorly characterized in pancreas may reflect ER stress or adaptive transcriptional responses in MFP.
**CEACAM1** Carcinoembryonic antigen-related cell adhesion molecule 1	glycoprotein implicated in cell adhesion and immune modulation	Top FC in MFP. Preserved expression in MFP may suggest intact epithelial and immune surveillance functions. Downregulated in many cancers, but in pancreatic ductal adenocarcinoma, CEACAM1 expression is typically upregulated
**KLF3** (Krüppel-like factor 3)	Transcription factor involved in inflammation and gene regulation; may influence cancer differentiation pathways.	Rarely studied in pancreas but linked to cancer differentiation pathways. May point to transcriptional reprogramming in MFP.
**GSN** (Gelsolin)	Regulates actin filament dynamics and has tumor suppressor functions.	Loss in PDAC is common, so its elevation in MFP is notable may be indicator of preserved or reactive cytoskeletal function in non-malignant tissue.
**MYH10** (Myosin heavy chain 10, non-muscle)	Myosin heavy chain—part of cytoskeletal remodeling, often linked to migration and mechanical adaptation.	High in MFP may indicate reparative or fibrotic response.
PDAC		
**NQO1** (NAD(P)H dehydrogenase [quinone] 1)	Antioxidant enzyme involved in detoxification and redox homeostasis.	Top FC in PDAC. Associated with oxidative stress resistance; often overexpressed in solid tumors including PDAC.
**TMEM205** (Transmembrane protein 205)	Known chemoresistance protein—upregulated in cisplatin-resistant tumors.	Top FC in PDAC. Its overexpression in PDAC is supported by transcriptomic and proteomic analyses, and it is considered a marker of poor prognosis and therapy resistance.
**HSD17B12** (Hydroxysteroid 17-beta dehydrogenase 12)	Enzyme involved in long-chain fatty acid elongation; linked to aggressive cancer metabolism and lipid biosynthesis.	Less explored in PDAC but may relate to metabolic rewiring.
**ECI1** (Enoyl-CoA delta isomerase 1)	Mitochondrial fatty acid oxidation enzyme; marker of metabolic shifts in cancer cells.	Upregulation in PDAC reflects a shift toward enhanced mitochondrial metabolism and is a marker of metabolic adaptation in cancer cells
**CMPK1** (Cytidine/uridine monophosphate kinase 1)	Nucleotide biosynthesis enzyme; associated with cell proliferation and chemotherapy response.	Clinically relevant for PDAC.
**SURF4** (Surfeit locus protein 4)	ER-Golgi transport protein; involved in secretion and trafficking of cancer-related proteins.	Supports increased secretory activity and protein trafficking, which are features of malignant transformation.
**HADH** (Hydroxyacyl-CoA dehydrogenase)	Mitochondrial fatty acid beta-oxidation enzyme; contributes to cancer metabolic reprogramming.	Suggests PDAC energy metabolism reprogramming.
**PDXDC1** (Pyridoxal dependent decarboxylase domain containing 1)	Enzyme potentially involved in vitamin B6-dependent reactions; may indicate metabolic plasticity.	Rarely studied but annotated for vitamin B6-dependent processes. May signal unexplored metabolic flexibility in PDAC.

Abbreviations: PDAC = pancreatic ductal adenocarcinoma, MFP = mass-forming chronic pancreati-tis, PPI = protein–protein interactions.

## Data Availability

The research data are available from the corresponding author upon reasonable request. The original mass spectrometry data presented in this study are openly available in the MassIVE repository at https://massive.ucsd.edu (accessed on 30 July 2025) under the accession number MSV000098984.

## Supplementary Materials

The following supporting information can be downloaded at: https://www.mdpi.com/article/10.3390/biomedicines13092199/s1, Figure S1: Functional classification and differential expression analysis results of differentially abundant proteins; Figure S2: STRING protein–protein interaction network for the differentially abundant proteins identified between MFP and PDAC and network clustering; Table S1. List of differentially abundant proteins (DAPs) in MFP vs. PDAC; Table S2. Characterization of differentially abundant proteins (DAPs) using the HPA Database; Table S3. ClueoGO/Cluepedia enrichment analysis results of DAPs; Table S4. STRING functional enrichment analysis results of DAPs.

## Abbreviations

The following abbreviations are used in this manuscript:

EUS-FNA	Endoscopic ultrasound fine needle aspiration
CP	Chronic pancreatitis
PDAC	Pancreatic adenocarcinoma
MFP	Mass-form chronic pancreatitis
PPI	Protein–protein interaction
