# Supplementary material for: Proteomic Profiling of EUS-FNA Samples Differentiates Pancreatic Adenocarcinoma from Mass-Forming Chronic Pancreatitis
Source: Biomedicines. 2025 Sep 8;13(9):2199. doi: 10.3390/biomedicines13092199 (PMC12467292; doi:10.3390/biomedicines13092199)
Supplement: Supplementary file 1 [file biomedicines-13-02199-s001.zip › Supplementary Figures 1_2.pdf]

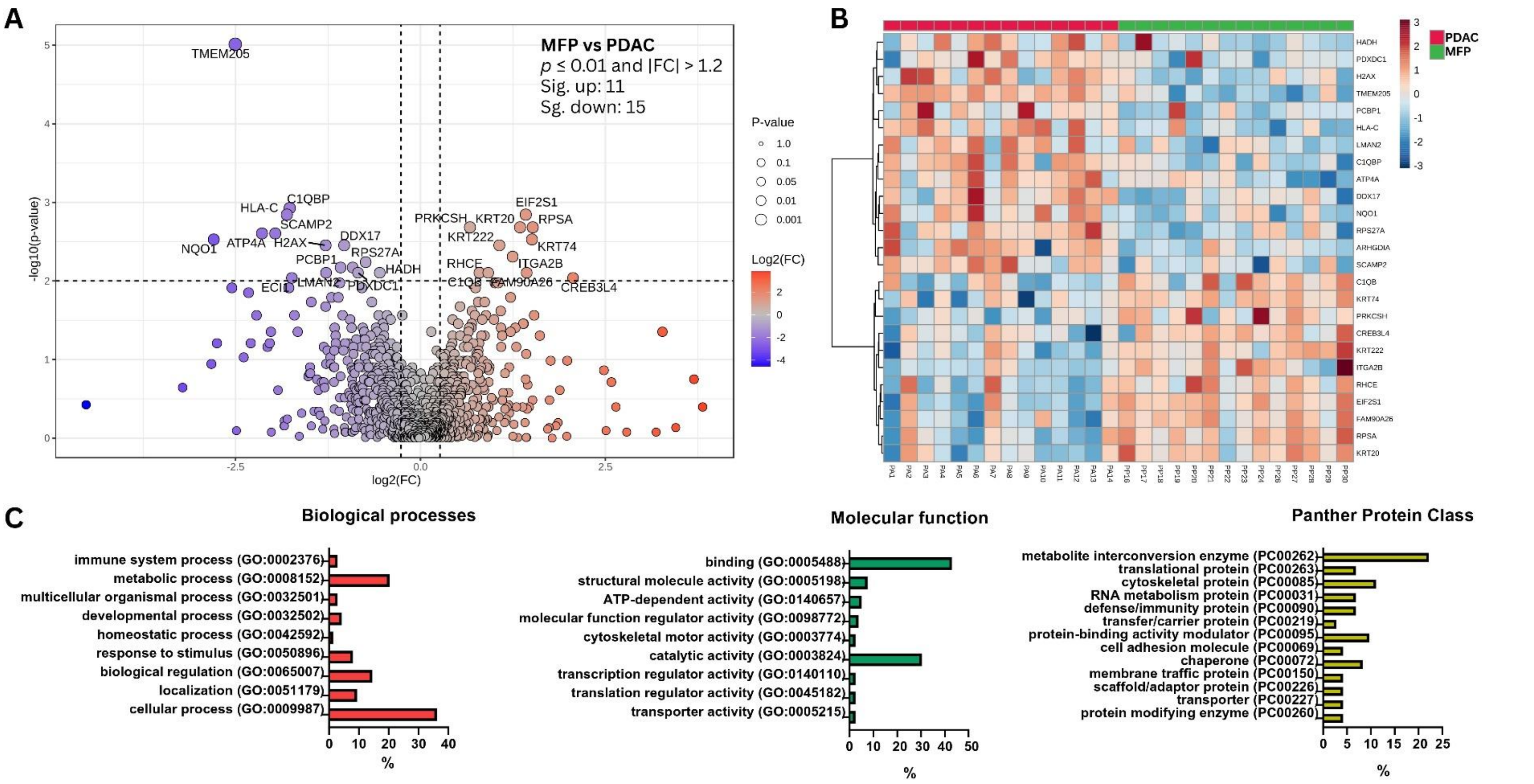

Figure S1. Functional classification and differential expression analysis results of differentially abundant proteins. **(A)** Volcano plot illustrating the differentially abundant proteins (*t-test*, independent unequal variance, p ≤ 0.01 and |FC| > 1.2) in the MFP group compared to the PDAC group. Red—DAPs with higher abundance in MFP; Blue—DAPs with higher abundance in PDAC; gray—proteins with no significantly different abundance. Gene names are provided for all significant DAPs; **(B)** Heatmap of differentially expressed proteins (unpaired t-test, unequal variance, p ≤ 0.01; Euclidean distance measure); **(C)** Functional classification analysis of DAPs using Panther online tool with GO (Biological Processes -BP, Molecular Function -MF) and Panther Protein Class ontologies.

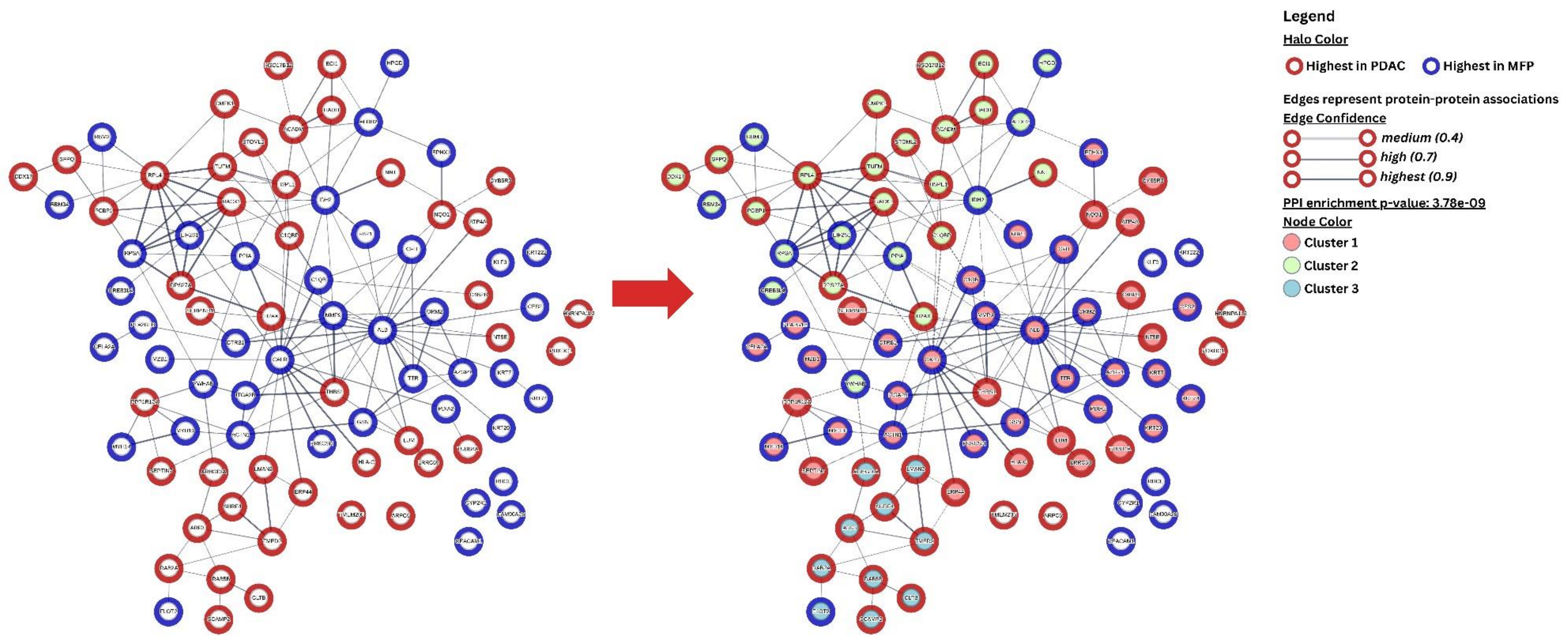

Figure S2. STRING protein–protein interaction network for the differentially abundant proteins identified between MFP and PDAC and network clustering using k-means clustering, k=3
